# Unique health identifiers for universal health coverage

**DOI:** 10.1186/s41043-019-0180-6

**Published:** 2019-10-18

**Authors:** Samuel Mills, Jane Kim Lee, Bahie Mary Rassekh, Martina Zorko Kodelja, Green Bae, Minah Kang, Supasit Pannarunothai, Boonchai Kijsanayotin

**Affiliations:** 1 World Bank Group, 1818 H Street, NW, Washington DC, 20433 USA; 2 Health Insurance Institute of Slovenia, Mikosiceva cesta 24, 1507 Ljubljana, Slovenia; 3 College of Pharmacy, Ewha Womans University, Seoul, South Korea; 4 Department of Public Administration, Ewha Womans University, Seoul, South Korea; 5 Centre for Health Equity Monitoring Foundation, 173/113, Moo 7, Phitsanulok-Nakhon Sawan Road, Thapho, Phitsanulok, 65000 Thailand; 6 Health System Research Institute, Ministry of Public Health, 88/39 Tiwanon 14 Road, Muang District, Nonthaburi, 11000 Thailand

**Keywords:** Unique health identifier, Universal health coverage, Unique identification number, Civil registration, Vital statistics, Electronic medical records

## Abstract

Identifying everyone residing in a country, especially the poor, is an indispensable part of pursuing universal health coverage (UHC). Having information on an individuals’ financial protection is also imperative for measuring the progress of UHC. This paper examines different ways of instituting a system of unique health identifiers that can lead toward achieving UHC, particularly in relation to utilizing universal civil registration and national unique identification number systems. Civil registration is a fundamental function of the government that establishes a legal identity for individuals and enables them to access essential public services. National unique identification numbers assigned at birth registration can further link their vital event information with data collected in different sectors, including in finance and health. Some countries use the national unique identification number as the unique health identifier, such as is done in South Korea and Thailand. In other countries, a unique health identifier is created in addition to the national unique identification number, but the two numbers are linked; Slovenia offers an example of this arrangement. The advantages and disadvantages of the system types are discussed in the paper. In either approach, linking the health system with the civil registration and national identity management systems contributed to advancing effective and efficient UHC programs in those countries.

## Background

Universal health coverage (UHC) is defined by the World Health Organization (WHO) as when “all people and communities can use the promotive, preventive, curative, rehabilitative and palliative health services they need, of sufficient quality to be effective, while also ensuring that the use of these services does not expose the user to financial hardship” [1]. UHC is fundamental to ending poverty and supporting individuals to enable them to manage their health and thus be able to contribute to their society. Recognizing its essential role in sustainable development, Sustainable Development Goal (SDG) Target 3.8 urges countries to “achieve UHC, including financial risk protection, access to quality essential health-care services and access to safe, effective, quality and affordable essential medicines and vaccines for all.” To provide UHC for *all*, countries need to first be able to identify everyone residing in their country, especially the poor. Therefore, having unique identifiers of individuals living in the country is a necessary component of achieving UHC [2].

Unique identifiers are also important in monitoring the progress of UHC, which is measured by two components: (i) the proportion of the population that has access to essential quality health care services and (ii) the proportion of the population for which health costs take up a large part of household expenditures [3]. To calculate these proportions, in addition to knowing the total number of the population (denominator), countries need to have disaggregated data of the entire population (for example, by age, sex, and residence). The best source of this type of universal and continuous disaggregated data pertaining to the population is the civil registration and vital statistics (CRVS) system that is linked to the national identity management system, whose data linkage via the unique identifier further enables access to data in other functional registers, including those of the health and finance sectors. There are also other (less comprehensive) ways of obtaining these data when CRVS is not yet possible, such as through population surveys, censuses, administrative systems, and surveillance systems [4].

Countries use various methods to uniquely identify individuals for providing health care [5]: (a) some countries assign a national unique health identifier (UHI) to each individual for health care, in addition to having a unique identification number (UIN) assigned to each individual via the national identity management system; (b) in other countries, without creating another unique number specifically for health, the UIN is used for health purposes as well; and (c) in others, individuals are assigned a UHI without having a UIN (see Table 1). Yet, there still remain some low-income countries that do not have either the UIN or the UHI at the national level, and where different health facilities generate their own patient numbers for administrative purposes but with limited utility for linking data with other health systems.

**Table 1:** Criteria for selecting the countries for comparing the institution of national unique health identifier numbers

	Slovenia	South Korea	Thailand	England
Achieved universal health coverage	Yes	Yes	Yes	Yes
Income classification when UHC was achieved	High	High	Lower-middle*	High
Has in place a functioning civil registration system	Yes	Yes	Yes	Yes
Assigns a national unique identification number (UIN)	Yes	Yes	Yes	No
Assigns a national unique health identifier (UHI)	Yes; UIN and UHI are highly linked	No; UIN is used as UHI	No; UIN is used as UHI	Yes

*Thailand achieved UHC in 2001 when it was still classified as a lower-middle-income country. Thailand became an upper-middle-income country in 2011

The present paper explains the importance of governments being able to identify individuals in their population in order to provide and assess the extent of UHC. It includes four case studies which demonstrate the methods that three high-income and one lower-middle-income countries used to allocate unique identifiers for health, and their advantages and disadvantages in implementation. The purpose of the paper is to provide examples of systems that other governments could use to achieve and monitor the progress of UHC using UHIs.

### Country examples of employing unique health identifiers

In countries where private health care is predominant, such as in the USA, different health care service organizations create their own patient identification numbers, making it difficult to connect one individual’s health records from different health care providers. To connect health records from disparate health information systems without a national UHI, one must perform statistical matching based on multiple patient characteristics (e.g., name, date of birth, and social security number), a method which has been analyzed to be less accurate and less cost-effective than using a UHI system [6]. Hence, in the USA, the Health Insurance Portability and Accountability Act of 1996 (HIPAA) [7, 8] mandated the Secretary of Health and Human Services to develop standards for issuing national UHIs to individuals, which is currently under works [9]. UNAIDS also offers guidance for countries adopting national health identifiers [10].

Among the countries that create UHIs, the level of centralization and efficiency of the process of UHI assignment also varies, especially depending on the level of Internet and system connectivity between the different levels of government offices, which often correlates with the country’s level of per capita income. Ideally, when the Internet and system connectivity allow lower-level local government authorities to have online access to the central database, the processes of verifying and managing existing UHIs and creating new UHIs can be done efficiently with a relatively lower risk of creating more than one UHI per person. However, when UHIs have to be created offline due to lack of Internet and/or system connectivity in low-income countries, the procedures for verifying identities and assigning new UHIs between the central-level and local offices require more manual efforts and therefore take longer to process. There are roughly two ways of creating UHIs offline (Dharwadker and Mills: Use of Unique Health Identifiers in Universal Health Coverage Programs or Health Insurance Schemes, unpublished): (i) local offices first submit new enrollment forms to the central office, where health identification (ID) cards with newly assigned UHIs are printed and sent back in batches to the local offices; and (ii) the central office distributes rolls of preprinted machine-readable UHI stickers to local healthcare or insurance offices for them to assign and affix new UHI stickers on ID cards at the time of enrollment and send back the duplicate stickers to the central office, so that the information can be entered into the central system. This second offline method has the benefits of providing UHIs to the beneficiaries in a faster manner and requiring fewer return visits to the office and therefore enables faster health service provision, which can be especially helpful for people living in remote areas; however, it increases the risk of error and fraud, such as if someone tries to apply for a new UHI from multiple local offices during the same period. Guidance on developing and implementing national health identifiers in resource-limited countries is available by Beck and colleagues [11].

## Methods

Among the countries that use national UHIs to manage national health information networks, the present paper illustrates case studies from four countries to compare the different methods of managing the UHI system. The four countries are Slovenia [5], South Korea [12], Thailand [13], and England (Dharwadker and Mills: Use of Unique Health Identifiers in Universal Health Coverage Programs or Health Insurance Schemes, unpublished); the information for each of these four countries is mainly drawn from larger case studies. The criteria for selecting these countries are shown in Table 1. First, all the country examples are based on countries with strong national health services or comprehensive insurance plans that have resulted in the country achieving UHC. Second, the authors included an example of a country that achieved UHC when it was still a lower-middle-income country. Third, all the demonstrated countries have a functional civil registration system in place, because robust civil registration systems provide the foundation for reliable and up-to-date identity authentication. Fourth, the main variance for comparison depends on how the country assigns a national UHI, with or without the presence of a national UIN, and how the two numbers are reconciled. Slovenia assigns both UIN and UHI and links them, while South Korea and Thailand use the national UIN in the health sector as UHI. In England, only UHI is assigned without a national UIN. Also, except for Slovenia, which has a population size of about 2 million people [14], the other three countries are similar in their population sizes, ranging between 51 and 69 million people [15–17].

### Slovenia: both UIN and UHI are used and the two numbers are linked

Slovenia is a high-income country located in the Europe and Central Asia region with a population size of about two million people [14]. Slovenia uses both UIN and UHI, and the two numbers are highly linked by the central population register (CPR). Though the process of creating this link and data consolidation was lengthy, it yields myriads of benefits today. The process starts with birth registration. When a baby is born, the birth is registered by the health personnel electronically at the hospital before the family is discharged. This birth registration data from the civil register is then automatically sent to the CPR which creates a UIN for the newborn. Data from the CPR is then automatically shared with the Health Insurance Institute of Slovenia and the Financial Administration of the Republic of Slovenia which create a UHI and a tax number, respectively. Once the UHI is created, the newborn is added as a beneficiary in the national Compulsory Health Insurance (CHI) scheme, which covers 100% of the population having permanent residency in the country. Although the current CHI scheme was established in 1992, the inception of social health insurance in Slovenia dates back to 1896.

Today, the health system in Slovenia offers many services electronically to its residents, made possible by the use of national health identifiers that can connect various health care services nationwide. The government operates an online portal through which the insured can access their health information and manage a significant portion of their health care activities. Individuals can make appointments with their health care providers through eAppointment and look up their medical records, such as laboratory test results and physician notes, enabling the individuals to be better informed and take ownership of their health. The insured persons can view their claims and billing information online as well, supporting transparency and better financial management. Each insured person receives an insurance card containing a digital chip (mailed to the house) that can be used as a key to accessing health services. In case the original insurance card is lost, stolen, or damaged, individuals can print out a temporary insurance certificate from the online portal to continue their care and submit an online request for a replacement insurance card to be mailed to the house. These efficient online services that prevent a disruption of service lessen the chance of an individual having to make out-of-pocket payments for health care services.

Health care providers also benefit from using the online health portal, which is powered by the national health identifiers connecting wide-ranging health service utilization information. All physicians in the country can look up a patient’s comprehensive medical history, including medications history, to provide continuous care. Prescription for medications can be electronically transmitted to pharmacies, and patients can pick up their medications from any pharmacy in the country. This ePrescription service prevents errors that can occur from bad handwriting or the loss of paper prescriptions, thus increasing patient safety, in addition to being convenient. Similarly, with eReferral, physicians can refer patients to another doctor, including those at secondary and tertiary healthcare levels, electronically through the web portal. These innovations in health care processes lessen the amount of manual administrative tasks that health care providers have to perform, which saves time and money.

These comprehensive online services are possible largely because of the connectivity of data from multiple databases. As previously mentioned, the three unique numbers, namely the UHI, UIN, and tax number of each individual, are used for various services across multiple sectors throughout the individual’s life, such as for travel, health, tax, income, and employment. Data collected through these transactions can be linked and retrieved using the unique identifiers, and these integrated data can better support monitoring and evaluating of public policies and programs. An example can be drawn from the government’s recent efforts with the colorectal cancer screening program. Recognizing that colorectal cancer is the second most common cancer in Slovenia despite the availability of effective prevention methods, the government proactively carried out free early-detection screening among the target population. In this process, data from the CPR provided the requisite information to select the target population, based on age, and medical history, among others. Through this program, from January to December 2017, out of 302,819 people invited, 168,823 submitted suitable stool samples for analysis and 10,536 showed positive results. This was followed by 10,995 colonoscopies which detected 214 cases of colorectal cancer and 2429 people with advanced adenoma, which presents a higher risk of cancer [5]. In this way, Slovenia utilized the UHI and CPR to efficiently carry out public health prevention and treatment programs.

To implement such extensive electronic transactions and data sharing using sensitive data, there are several measures in place to ensure data protection and privacy. First, The Central Population Register Act [18] was established in 2006 to protect personal information and to regulate the flow and use of data. Second, to safeguard access to the information in the chip insurance card, health care professionals are given card readers and their own health professional cards which are password-protected. Both the patient card and health professional card must be inserted into the card reader at the same time, plus a passcode, to access data. Health care personnel, depending on their roles, have a varying degree of access to patient information. For example, while physicians may have full access to patient medical history, the administrative staff, whose main role is scheduling appointments and handling billing, may only be able to view limited clinical information. Researchers, on the other hand, would have access to de-identified data.

Analysis of the electronic civil registration and health data provides information for the detection and prevention of fraud. Connection with the civil registration data prevents false registration for health insurance because identity verification can be done in real time with access to the central database. Review of electronic medication records can provide accountability and prevent fraud against prescribing medications for wrong indications, wrong dosage, or in wrong combinations. Similarly, analyzing the billing information can also reveal fraudulent claims and enhance patient safety, for example, by detecting services billed that are inappropriate for the patient’s age or gender.

### South Korea: UINs are used as UHIs

South Korea is a high-income country located in East Asia with about 51 million population [15]. The CRVS system in South Korea was developed in the 1960s and is comprised of three parts: Family Relationship Registration (FRR), Resident Registration (RR), and vital statistics. When birth registration is done as part of FRR, the system creates an RR number for the RR system which mainly manages the population’s residential status and migration. Moreover, the RR number is widely used for accessing services in other sectors as well, including as a UHI to access benefits through the national health insurance (NHI) system [12]. The NHI is a social insurance program with universal coverage that requires enrollment by all citizens and medical facilities. While the insurance premium cost is determined based on the insured’s income, the coverage is uniform across the population. Individuals’ RR numbers are used to link information stored in different databases, such as those in relation to health, income, property, tax, and family relationship, to calculate the contribution rate. The RR card is issued to all citizens 17 years of age and older, and it can be presented to access health care without having to carry a separate health insurance card. Online services are available to individuals as well, accessible by entering their RR numbers. On the medical record-sharing web portal, people can view their medical records and confirm appointments and referrals. Health care providers’ RR numbers are also linked with their medical license numbers and medical facility numbers. This information is used to process reimbursements for services provided and, in some cases, to provide performance-based incentives to health care providers. The RR numbers are also used by the government to compile the necessary information to determine who needs medical care assistance (for low-income citizens) and/or extended health coverage to prevent the occurrence of catastrophic health expenses paid by the population. Extended health coverage is provided to those who have been diagnosed with one of the four major serious diseases (cancer, heart disease, brain disease, and rare and incurable diseases) for covering high-priced drugs, treatments, tests, and so forth. These efforts are in line with the aim of UHC to prevent financial impact from catastrophic health expenses.

In the area of disease prevention and health promotion programs, the RR number plays an essential role. Managing vaccination programs is one example. All vaccination records are linked via the RR number between the Korea Centers for Disease Control and Prevention (KCDC); the National Health Insurance Service (NHIS), which has vaccination records for infants and toddlers; local public health centers and private medical facilities that provide vaccinations; the Ministry of Health and Welfare’s social welfare integrated information system and integrated child care management system; and the Ministry of Education’s national education information system that is used by schools. This integrated data can be accessed by medical professionals to avoid giving double vaccinations. Individuals can also look up and manage their own vaccination history on the KCDC website. Another example is a meal aid program for undernourished children provided by local government offices. In choosing the program recipient, local offices use the RR numbers to look up information on age, household income, health insurance premium, a child’s relationship with the guardian (especially to find single-parent households), and whether a child has disabilities. Similar information is accessed by local public health centers via RR numbers to choose recipients for the Nutri Plus Program for infants under 6 months old, pregnant women, and new mothers. For this program, RR numbers are used to verify the relationship between the mother and child and to look up information on the number of household members, health insurance premium, income level, and so forth. Once chosen, recipients are provided with nutrition education, nutrient-rich foods, regular nutrition check-ups, and counseling. The RR numbers are also used to track and record the nutrition status of the recipients throughout the program for evaluation purposes; the recipients’ consent is obtained prior to collecting personal information. In a like manner, local public health centers use the RR numbers in the process of selecting recipients for several other health promotion programs. These include maternal and child health programs, prenatal and postpartum care support services, adolescent pregnancy and childbirth medical expense support programs, infertility treatment support programs, and newborn hearing screenings, to name a few. For many of these programs, the RR numbers are associated with voucher cards, and program information and materials are sent to the address associated with the recipient’s RR number.

The RR numbers are also well integrated and utilized for disease treatment and management. For controlling and limiting the spread of contagious diseases for public health, if the severity of the condition is regarded as being potentially epidemic, medical facilities and other infectious disease surveillance agencies are required by law to report the infected person’s personal information, including the RR number, to the KCDC. Reporting the RR number in addition to the name helps prevent double registrations and better facilitate administrative tasks, such as processing insurance claims, receiving paid leave from employment, and receiving livelihood assistance for the duration of being quarantined. Tuberculosis and HIV/AIDS patients can receive free treatment as part of the NHI plan benefits if they register with their RR numbers. It is noteworthy to mention the method of managing chronic diseases, in particular hypertension and diabetes, using the RR number and technology in South Korea. People who subscribe to the NHIS’ health information website can receive customized health information. For those with hypertension and diabetes, as a pilot program, they can use the website or a mobile app to regularly send in their blood pressure and blood sugar measurements, and a health care provider reviews the data remotely. When a management plan has been posted online, the subscriber receives a notification, and phone counseling with a health care provider is available, if needed.

In 2017, the Enforcement Decree of the Medical Care Assistance Act required standardization of electronic health records to facilitate sharing of information across medical facilities. Following this decree, a health information exchange system was established to share electronic medical records, including information such as diagnosis, drugs, laboratory and radiological test results, referrals, and transcripts. Even in large hospitals that create their own patient numbers, RR numbers are still collected and entered into the electronic medical records to support the data-sharing activities.

The main challenge of using the RR number for health care is its threats to privacy and protection of personal information. Due to the wide-ranging uses, Korean residents use their RR number for various public and private services every day. Data leakage on the population’s RR numbers at any service point could lead to serious violation of sensitive personal data. The history of health data leakage in Korea [19–22], such as hospital website intrusions, and hospitals’ illegal use of patient data for research purposes have been challenges the country continues to address and strengthen.

### Thailand: integrated civil registration and UIN system contributed to achieving UHC as a lower-middle-income country

Thailand is located in the East Asia and Pacific region and has a total population of about 69 million people [17]. With significant social and economic growth in the last four decades, Thailand in 2011 moved from being a low-income country to becoming an upper-middle income country [17]. Before it became an upper-middle income country, Thailand achieved UHC as a lower-middle-income country in 2001. Among the many factors that contributed to achieving UHC, having an integrated civil registration and UIN system played an essential role in compiling a list of beneficiaries to enroll into the national health insurance scheme [13]. The civil registration system in Thailand moved from a paper-based system to a computerized system in 1982, facilitated by first assigning a UIN, called a personal identification number (PID), to each Thai citizen, then entering their information into the central population database. The civil registration system was centralized, with the district and provincial databases connected online to the central database. Along with the improvements in civil registration, vital statistics also dramatically began to improve once birth and death registration data from the civil register automatically fed into the Ministry of Public Health’s vital statistics database starting in 1996.

When a baby is born, either the hospital or the village head provides a birth notification form to the child’s parents, who then take the notification form to the district registration office along with his/her proof of identity to register the birth of the child. At the district registration office, the registrar can verify the submitted information against the central database to register the child, issue a birth certificate, and assign a PID to the child. From that point on, the PID is used to identify whether the child is a Thai citizen, and if he/she is, the child is automatically enrolled into the national health insurance scheme. Because free health care and government child support are provided to Thai citizens with a PID, this incentive has increased the demand for birth registration.

PIDs are used for more than enrolment into the insurance scheme. Now, electronic medical records are used at all hospitals. Even though each hospital’s health care information system creates its own patient identification number, this number is linked to the individual’s national PID. Then, health care professionals use the PIDs to check patient eligibility, track health care services provided, and process claims, among other activities. PIDs ideally make electronic medical records sharable among health care providers. Currently, many projects are being piloted to implement sending of referrals electronically. Thai citizens use chip-containing citizen identification cards to access health services.

Similar to the challenges that South Korea faces, also in Thailand, the risk of data leakage and identity theft has increased with the introduction of the wide use of a national unique identifier. In an effort to address this problem, Thai government is striving to build a national single-point digital identity platform so that identity verification is done on only one government platform to reduce the exposure and misuse of personal information.

### England: UHIs are assigned without national UINs

In some countries, UHC has been achieved with the employment of UHIs without assigning national UINs. England is an exemplary case. This European country currently has about 66 million people living in the territory, and it has been providing universal health care through the National Health Service (NHS) since 1948. In the absence of a national UIN system, England assigns a 10-digit UHI called the NHS number to its residents. Almost all medical services, including medications prescribed during a hospital visit, are covered by the state-funded insurance scheme, while a few services and medications at pharmacies charge a nominal patient contribution. Only about 11% of the population purchase a private health insurance [23]. Although individuals are not denied health care services if they do not have an NHS number, the NHS number enables patients to look up their electronic medical records (for general practice services), schedule appointments with their general practitioners, and order prescriptions online through the NHS website [24]. Electronic processes are also used in the way health care providers are paid for the services they deliver; data are extracted automatically from electronic medical records to calculate their reimbursements [23]. Electronic medical records are not yet connected among all health care providers, but all NHS processes are projected to be handled electronically by 2023.

Separately from the health sector, the General Register Office, a part of Her Majesty’s Passport Office, oversees the registration of all births, deaths, marriages, civil partnership, adoption, and stillbirth occurring in England and Wales [25]. The national archive of all births, marriages, and deaths since 1837 are maintained by the Office. In this way, in the absence of a national UIN, England is able to separately operate successfully both civil registration and universal health care with the use of a UHI.

### Pros and cons of the different methods for assigning a national UHI

Table 2 presents the advantages and disadvantages of each of the methods of assigning a national UHI. First, when the national UIN is used as UHI in the health sector, it facilitates sharing information across several sectors, as necessary, provided that data privacy and confidentiality are secure. Another advantage of using an existing national identifier is that the infrastructure and personnel who are knowledgeable in the identity management processes are already in place. It would cost less to increase the existing infrastructure and staff size, rather than to build a new parallel system. On the flip side, with the increase in scope and use of the national UIN with a larger number of institutions and personnel handling this sensitive information, the risk of potential data breach also increases, as well as the number of people who will attempt identity theft and fraud to benefit from different services. In countries where civil registration is weak, not all health service users may have proof of national identity or legal identity (such as a birth certificate) and pose barriers or delays in receiving health services.

**Table 2:** Pros and cons of the different methods for instituting a national UHI

Method of assigning a national unique health identifier	Pros	Cons
(1) UIN is used as UHI	- Can connect with other systems, beyond the health sector - Lower cost in infrastructure and human resources	- Greater potential for data breach and fraud - Not all health service users may have proof of national ID
(2) UIN and UHI are both used and linked to each other	- Can connect with other systems, beyond the health sector - Lower risk of misuse of the national UIN	- Higher cost of maintaining infrastructure and human resources for having two identity management systems
(3) No UIN; Only UHI is assigned	- Superior confidentiality comes with limited scope and use of the UHI	- UHI is used for health purposes only and not for other sectors

When both UIN and UHI are used and securely linked to each other, this setup can enable linking an individual’s health information with data about that person collected and stored in other sectors. Moreover, because the UHI does not show the actual UIN to many of its users and internal personnel who process them, it reduces the risk of exposing the UIN and making other personal information vulnerable for data breach or misuse. Nevertheless, the system needs to be able to identify a person in several different ways. For example, if a person forgets his/her UHI number, health service providers need to be able to identify the person using the UIN, with appropriate proof of identity. Having multiple identity management systems would cost more in terms of its infrastructure use and human resources, especially if the two systems are housed by different agencies, compared to using one unique identifier for multiple uses.

When no UIN exists and only national UHIs are assigned, its relatively limited use only in the health sector increases the confidentiality of patient data, since the UHI will not provide access to other information such as an individual’s financial information. However, it also means that the UHI is only useful in the health sector and not for accessing or analyzing information in other sectors.

Asian Development Bank (ADB)’s situational analysis of national UHIs in Cambodia, the Lao People’s Democratic Republic (PDR), and Myanmar provides insight into common problems and barriers that countries face when establishing UHIs [26]. Especially in low- and middle-income countries, the lack of information and communications technology, trained personnel, appropriate legal framework for protecting data privacy, and institutional framework for multisectoral coordination and interoperability, as well as donor’s disease- or program-specific approach to collecting data, often causes bottlenecks in successfully using national UHIs to consolidate disparate health information systems.

## Conclusion

A robust civil registration system that is integrated with UINs contributes to implementing effective and efficient UHC programs. Civil registration, by definition, is universal and continuous. It identifies and establishes a legal identity for every person in the country, which is also fundamental in order to execute UHC, to provide essential health care for all. UINs further link the unique identities to their financial and social information collected by other sectors. Access to this information is critical for measuring UHC, because UHC aims to provide essential health care services to all people without causing financial harm from health expenditures. As we have seen from the country examples highlighted above, there are different ways of incorporating UINs into the national health system. Separate UHIs can be created in addition to UINs while the two numbers are linked and allow efficient data exchange, as is the case in Slovenia. UINs can also be used as the UHIs, while being used as a key to accessing other varieties of public and private sector services, which is being done in South Korea. This utilization of civil registration and UIN data for achieving UHC has been possible in low-income settings as well, as exemplified by Thailand in a process that culminated in 2001 as a lower-middle-income country at the time. Authors discussed the advantages and disadvantages of these system types in implementation. Countries working toward UHC should consider the benefits of an integrated civil registration and UIN system and leverage its utilities for improving UHC programs.

## Data Availability

Not applicable.

## Abbreviations

CPR: Central population register
CRVS: Civil registration and vital statistics
FRR: Family relationship registration
ID: Identification
KCDC: Korea Centers for Disease Control and Prevention
NHI: National health insurance
NHIS: National Health Insurance Service
NHS: National Health Service
PID: Personal identification number
RR: Resident registration
SDG: Sustainable Development Goals
UHC: Universal health coverage
UHI: Unique health identifier
UIN: Unique identification number
WHO: World Health Organization
